# Legionnaires’ Disease Outbreak Caused by Endemic Strain of *Legionella pneumophila*, New York, New York, USA, 2015

**DOI:** 10.3201/eid2311.170308

**Published:** 2017-11

**Authors:** Pascal Lapierre, Elizabeth Nazarian, Yan Zhu, Danielle Wroblewski, Amy Saylors, Teresa Passaretti, Scott Hughes, Anthony Tran, Ying Lin, John Kornblum, Shatavia S. Morrison, Jeffrey W. Mercante, Robert Fitzhenry, Don Weiss, Brian H. Raphael, Jay K. Varma, Howard A. Zucker, Jennifer L. Rakeman, Kimberlee A. Musser

**Affiliations:** Wadsworth Center, Albany, New York, USA (P. Lapierre, E. Nazarian, Y. Zhu, D. Wroblewski, A. Saylors, T. Passaretti, H.A. Zucker, K.A. Musser);; New York City Department of Health and Mental Hygiene, New York, New York, USA (S. Hughes, A. Tran, Y. Lin, J. Kornblum, R. Fitzhenry, D. Weiss, J.K. Varma, J.L. Rakeman);; Centers for Disease Control and Prevention, Atlanta, Georgia, USA (S.S. Morrison, J.W. Mercante, B.H. Raphael);; New York State Health Commissioner, Albany (H.A. Zucker)

**Keywords:** Legionnaires’ disease, legionellosis, Legionella, Legionella pneumophila, bacteria, endemic strain, whole-genome sequencing, PCR, pulsed-field gel electrophoresis, outbreak, outbreak investigation, water, cooling towers, New York City, Bronx, New York, United States

## Abstract

During the summer of 2015, New York, New York, USA, had one of the largest and deadliest outbreaks of Legionnaires’ disease in the history of the United States. A total of 138 cases and 16 deaths were linked to a single cooling tower in the South Bronx. Analysis of environmental samples and clinical isolates showed that sporadic cases of legionellosis before, during, and after the outbreak could be traced to a slowly evolving, single-ancestor strain. Detection of an ostensibly virulent *Legionella* strain endemic to the Bronx community suggests potential risk for future cases of legionellosis in the area. The genetic homogeneity of the *Legionella* population in this area might complicate investigations and interpretations of future outbreaks of Legionnaires’ disease.

*Legionella* spp. are ubiquitous in nature, live in soil and water, and frequently inhabit human-made water distribution systems, hot water tanks, decorative fountains, and cooling towers (*1*,*2*). Persons with underlying health conditions, such as chronic lung disease, or those with compromised immunity are at increased risk for contracting Legionnaires’ disease (LD) (also referred to as legionellosis). Signs and symptoms typically include fever, cough, and chest pain; LD is fatal in ≈5%–10% of cases (*3*,*4*). Transmission of *Legionella pneumophila* is believed to occur mainly through exposure to contaminated aerosols and not from other infected persons; to date, only 1 case of human-to-human transmission has been documented (*4*,*5*).

LD was initially detected in 1976, when an outbreak of illness occurred during a meeting of the American Legion in Philadelphia, Pennsylvania, USA; 221 cases were identified, and 34 infected persons died (*6*). The outbreak, which remains the largest community-associated outbreak of LD in United States, was later linked to the cooling system of the hosting hotel, and a bacterium classified as *L. pneumophila* serogroup 1 was subsequently isolated from 4 persons (*7*,*8*).

In the summer of 2015, a large community-associated LD outbreak affected persons who resided or traveled through a large area in the South Bronx region of New York, New York, USA. During July 2–August 3, a total of 138 adults with LD were linked to the outbreak; 128 patients required hospitalization, and 16 deaths occurred (Figure 1). A joint laboratory investigation to find the source of this outbreak was performed by the New York City Department of Health and Mental Hygiene and the Public Health Laboratory (NYC PHL), the Wadsworth Center (WC) of the New York State Department of Health (Albany, NY, USA), and the Centers for Disease Control and Prevention (CDC; Atlanta, GA, USA).

**Figure 1:**
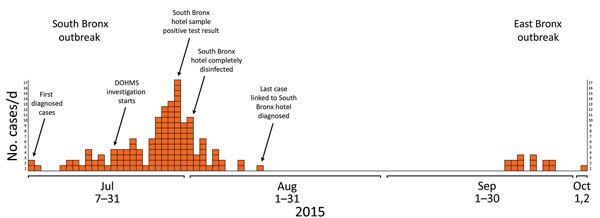
Legionnaires’ disease outbreak caused by an endemic strain of *Legionella pneumophila*, New York City, New York, USA, 2015. Timeline shows all diagnosed cases linked to the July 2015 South Bronx and August 2015 East Bronx outbreaks. Each orange square represents the time at which a person was given a diagnosis of the disease. Annotations of some of the key actions taken by the authorities are listed above their corresponding days. DOHMS, Department of Health and Mental Hygiene.

Pulsed-field gel electrophoresis (PFGE), real-time PCR, sequence-based typing (SBT), and whole-genome sequencing (WGS) were used to characterize human and environmental *L. pneumophila* isolates from the investigation. Epidemiologic data and water testing by PCR quickly led to identification of a cooling tower located on the roof of a South Bronx hotel as a potential source of this outbreak (*9*). However, *L. pneumophila* isolates recovered from a sample taken later during the outbreak from a homeless shelter located in the vicinity of the South Bronx hotel and others facilities within the outbreak zone were found to have PFGE and SBT patterns identical to that of the outbreak strain, raising the possibility that the South Bronx hotel might not have been the only source of an aerosolized *Legionella* species associated with cases of legionellosis. Our finding of highly related *L. pneumophila* isolates in multiple environmental samples and from past LD outbreaks suggests the presence of a potentially pathogenic endemic strain in the Bronx community.

## Methods

### Water Samples and Clinical Isolates

Initially, water and swab samples were collected by the New York City Department of Health and Mental Hygiene and split between the WC and the NYC PHL. Later in the outbreak, water and swab samples were also collected by the New York State Department of Health and submitted to WC. Samples were processed as described (*9*), except for a subset of samples, including swab samples and visibly complex samples, that were not concentrated by centrifugation but tested directly. Clinical isolates were received by the NYC PHL and forwarded to WC. A subset of water and clinical isolates was sent to CDC for SBT analysis or sequencing by using the RSII Platform (Pacific Biosciences, Menlo Park, CA, USA). SBT was performed according to the European Society of Clinical Microbiology and Infectious Diseases (Basel, Switzerland) Study Group for *Legionella* Infections Scheme (*10**,**11*).

### Extraction of DNA

Nucleic acid extraction was performed for water and swab samples by using a modified Masterpure DNA Isolation Kit procedure (Epicentre, Madison, WI, USA) (*12*). In brief, for each extraction, a 1.2–1.5 McFarland suspension of the isolate in sterile water was centrifuged for 10 min at 7,500 rpm. A volume of 950 μL of supernatant was removed, leaving 50 μL. A total of 300 μL of 2× tissue and cell lysis buffer containing 1.5 μL of proteinase K was then added to each sample. Each extraction incorporated a negative extraction control that consisted of 50 μL of sterile water. The DNA was resuspended in 100 μL of 10 mmol/L Tris. Concentrations of the DNA were quantified by using the Qubit ds DNA HS Assay Kit (Thermo Fisher Scientific, Waltham, MA, USA) according to the manufacturer’s instrctions, with the Qubit 2.0 fluorometer before WGS. Updates to the protocol include the addition of an internal inhibition control. 

### PCR Screening

We tested processed samples for *Legionella* DNA using real-time PCR with a newly validated and more comprehensive procedure than that previously published (*12*,*13*) to rapidly screen samples for prioritizing culture efforts. This assay detects and differentiates *Legionella* spp., *L. pneumophila*, and *L. pneumophila* serogroup 1 and uses an internal control to assess for inhibitory substances in the sample.

### Culture of Water Samples

Samples in which *L. pneumophila* serogroup 1 was detected were processed and cultured at WC and NYC PHL by using standard methods. Isolates were identified as *L. pneumophila* serogroup 1 by using direct fluorescent antibody testing or real-time PCR. All *L. pneumophila* serogroup 1 isolates were initially typed by using digestion with *Sfi*1 and pulsed-field gel electrophoresis (PFGE) as described (*14*).

### WGS

DNA sequencing was performed by using the MiSeq platform (Illumina, San Diego, CA, USA) at the WC Applied Genomic Technologies Core and the RSII platform (Pacific Biosciences) at CDC. Individual sample libraries were prepared by using a Nextera XT protocol (Illumina) for sequencing. PacBio-compatible libraries were constructed by using 8 μg of sheared genomic DNA (≈15 kb) prepared by using the SMRTbell Template Prep Kit 1.0 (product number [PN] 100–259–100; Pacific Biosciences) according to the manufacturer’s protocol (PN 100-092-800-06), the PacBio Binding Calculator version 2.3.11, the DNA Polymerase Binding Kit P6 version 2 (PN 100-372-700), and the MagBead Kit (PN 100-133600).

Sequencing runs were performed with a 2-kb DNA internal control (PN 100-356-500), 240-min movie time, and stage start with a DNA Sequencing Reagent Kit 4.0 (PN 100-356-400). Final library size was confirmed by using the Agilent Tapestation 2200 and the Genomic DNA ScreenTape (5067–5365 and 5067–5366). Hierarchical Genome Assembly Process version 3 was used to construct the complete *L. pneumophila* genome sequences (*15*). The expected genome size was set to 3.4 Mb and target genome coverage parameter was set to 15×. The minimum subread length value was adjusted to decrease genome coverage to the recommended 100×—150× for microbial genomes (*16*). Genome closure was performed by identifying and trimming nucleotide overlap at the ends of the single assembled contig sequences with Gepard version 1.3 (*17*), and the reformatted genome sequence was used as input for the RS-ReSequencing protocol in the SMRT analysis portal to construct the polished genome sequence.

To confirm nucleotide accuracy, we aligned paired-end Illumina data for each sequenced isolate to its respective PacBio polished sequence by using Bowtie version 2.1.0 (*18*). We used Samtools version 0.1.18 (*19*) and FreeBayes version 0.9.21 (*20*) to identify nucleotide discrepancies between the 2 types of data. We resolved any discrepancies with the Illumina dataset and used VCFtools version 0.1.11 (http://vcftools.sourceforge.net/) to construct the final consensus sequence by using both data types (*21*). We deposited the closed genome sequence of the South Bronx outbreak strain F4469 in GenBank (accession no. CP014760). All raw Illumina reads used in this study are available in BioProject (accession no. PRJNA345011) (https://www.ncbi.nlm.nih.gov/bioproject/).

### Bioinformatics Analysis

We mapped raw reads to the South Bronx outbreak strain F4469 by using BWA MEM version 0.7.5a-r405 (*22*). Single-nucleotide polymorphisms (SNPs) were called by using Samtools/BCFtools version 0.1.19–44428cd (*19*), a minimum of Q20 for mapping quality and basecall quality, 10× minimum depth, and 95% of allele read agreements. Positions where >1 of the samples were found to have a mutation was manually verified and used to build a SNP alignment. Positions with ambiguous calls in any of the samples were discarded. We imported the resulting alignment into PHYLOVIZ (*23*) and built a minimum spanning tree by using the GoeBURST full minimum spanning tree algorithm (https://github.com/apetkau/microbial-informatics-2014/tree/master/labs/mst). Presence of plasmids was verified by performing de novo assemblies of different isolates in SPAdes version 3.7.0 (*24*) and compared by using Mauve (*25*). Recombination events were determined by finding regions of enriched SNP density by using a probability density function calculation and Fastgear (*26*). Blast ring genome comparisons were made by using BRIG (*27*).

## Results

WC tested 289 cooling tower water samples from 183 cooling towers by real-time PCR during this outbreak investigation. A total of 162 (88.5%) cooling towers were positive for *Legionella* species DNA. *L. pneumophila* DNA was detected in 87 (47.5%) cooling towers; 52 (28.4%) cooling towers were positive *L. pneumophila* serogroup 1, and 21 (11.5%) showed negative or inconclusive results.

**Figure 2:**
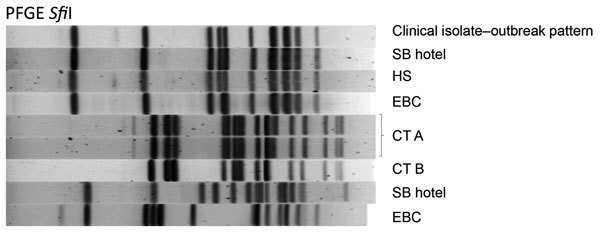
Pulsed-field gel electrophoresis (PFGE) of case-patient and environmental isolates from a Legionnaires’ disease outbreak caused by an endemic strain of *Legionella* *pneumophila*, New York City, New York, USA, 2015. One clinical isolate with the outbreak PFGE pattern (Clinical isolate−outbreak pattern) shows matches to cooling tower (CT A and CT B) isolates from a South Bronx hotel (SB), a homeless shelter (HS), and East Bronx College (EBC). Molecular typing patterns of *Legionella pneumophila* serogroup 1 isolates from CT from the SB hotel, HS, and EBC were indistinguishable from 26 clinical isolates associated with the Legionnaires’ disease cluster in the South Bronx. Samples that were not linked to the outbreak had major differences in patterns when compared with the outbreak pattern.

On the basis of the amount of DNA present, which was determined by the initial PCR screening, 26 cooling tower water samples were cultured during July 28–August 14, 2015. We identified 10 culture-positive cooling towers from which 15 *L. pneumophila* serogroup 1 isolates were subjected to PFGE. In addition, culture at NYC PHL identified isolates from a homeless shelter cooling tower that were identical by PFGE and included in this analysis. These *L. pneumophila* serogroup 1 PFGE results showed 7 PFGE patterns (Figure 2). Of these isolates, PFGE showed that those from the South Bronx hotel and the homeless shelter were identical to clinical isolates. SBT analysis showed that these isolates also had the same sequence type (i.e., 731).

WGS was used in real time during the course of the South Bronx LD outbreak as a confirmatory method and to provide additional insight on the source. The investigation also subsequently used WGS to help clarify whether the outbreak strain could have been present at other locations during the outbreak or at other times in the past. A total of 156 isolates of *L. pneumophila* serogroup 1 were available from culture performed at the WC, NYC PHL, and hospital laboratories (115 environmental and 41 clinical isolates from 26 patients, of which 35 were respiratory and 6 were postmortem specimens from 3 patients). These isolates were sequenced and analyzed by using an in-house bioinformatics pipeline developed at the WC.

**Figure 3:**
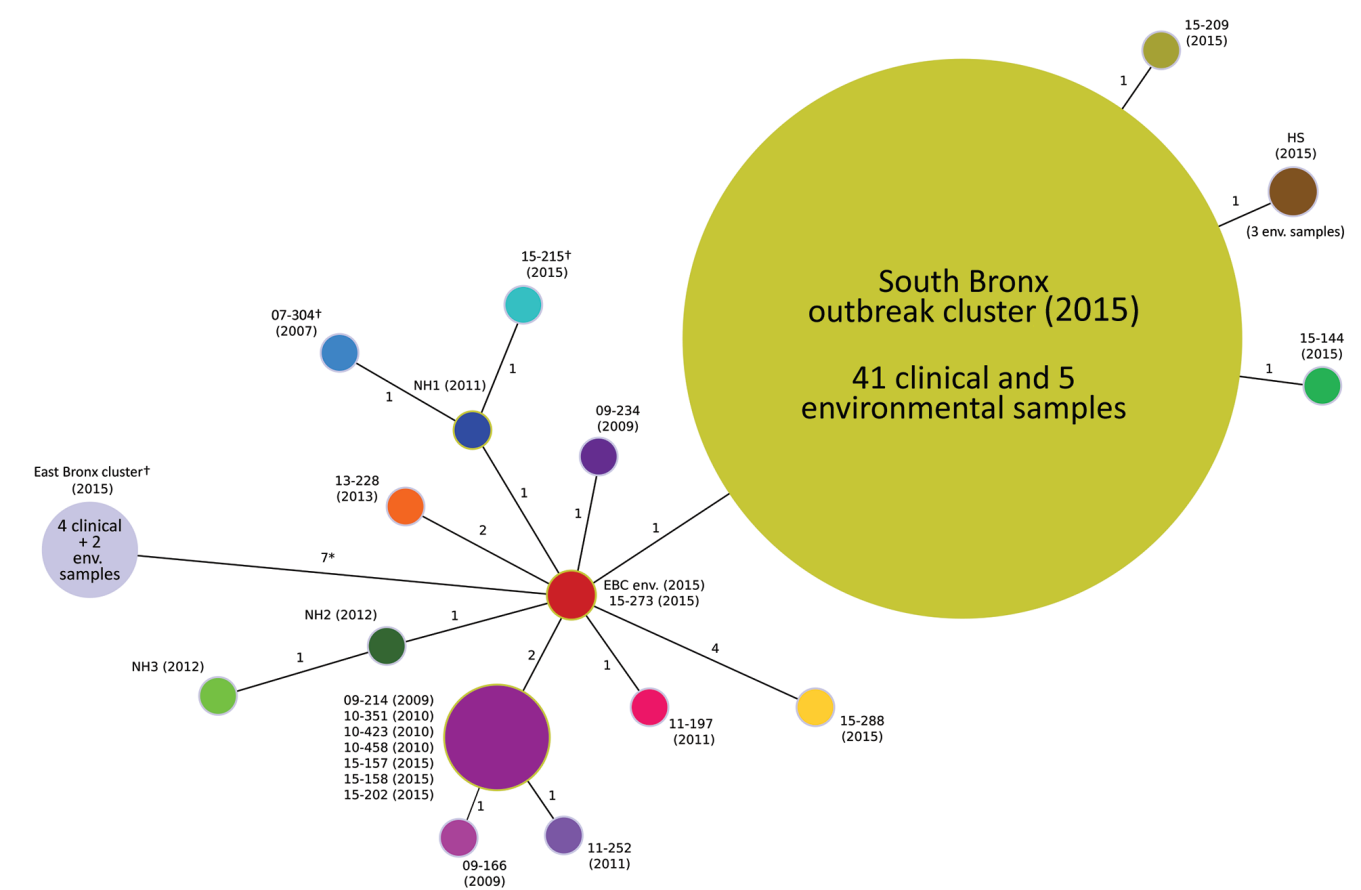
Minimum spanning tree of 77 isolates related to the 2015 South Bronx Legionnaires’ disease outbreak caused by an endemic strain of *Legionella* *pneumophila*, New York City, New York, USA, 2015. The tree was created by using single-nucleotide polymorphism (SNP) differences found across all isolates. Sizes of circles are proportional to number of isolates having identical genomic backgrounds, numbers adjacent to lines indicate number of polymorphism differences between each node, and numbers in parentheses indicate years. Strains 07-304 and 15-215 contain the same plasmid and differ greatly from the plasmid present in all East Bronx isolates. These plasmids have only partial identity with known plasmids in other *Legionellaceae*. *East Bronx outbreak samples contain 1,030 additional SNP differences caused by the presence of suspected homologous recombination events that were omitted from the final SNP analysis; †Plasmids are present in these isolates. EBC, East Bronx college; env., environmental; HS, homeless shelter; NH, nursing home.

Most (106/115) of the environmental *L. pneumophila* serogroup 1 isolates sequenced did not closely match any of the clinical *L. pneumophila* serogroup 1 isolates suspected to be part of this outbreak, and differed by several thousand SNPs over the 3.4-Mb genome of the South Bronx hotel strain F4469 used as a reference. Five *L. pneumophila* serogroup 1 isolates recovered from the South Bronx hotel and 41 clinical *L. pneumophila* serogroup 1 isolates from 26 patients linked to this outbreak were identical (no SNP differences among them) (Figure 3). Eight other *L. pneumophila* serogroup 1 clinical isolates (15–144, 15–157, 15–158, 15–202, 15–209, 15–215, 15–273, and 15–288) obtained during the same outbreak period had the same PFGE and SBT types as the outbreak isolates. However, these 8 isolates did not meet the epidemiologic case definition (*9*), and WGS showed that they contained 1–5 SNP differences compared with the South Bronx hotel isolate.

Four environmental isolates (3 isolates from the same homeless shelter and 1 from an East Bronx College) obtained during the investigation of the South Bronx outbreak (Figure 2) were nearly identical to the South Bronx hotel isolate, each differed by only 1 or 2 SNPs from the South Bronx hotel isolate and from one another. All 3 isolates from the homeless shelter had the same unique SNP that was absent from all clinical and environmental isolates linked to the South Bronx hotel. Moreover, the East Bronx College isolate, which was obtained from a site several kilometers from the South Bronx hotel, was identical by WGS to 1 South Bronx clinical isolate (15–273). Five other clinical isolates (15–288, 15–215, 15–157, 15–158, and 15–202) had SNP profiles that were closer to the isolate obtained from the East Bronx College than to the isolate obtained from the South Bronx hotel. Together, these observations suggest that 1) the South Bronx hotel cooling tower, and no other cooling towers, was most likely the source of the South Bronx outbreak; and 2) cases not epidemiologically linked with the outbreak might have originated from other environmental sources.

We also completed WGS for 10 historical clinical isolates of *L. pneumophila* serogroup 1 DNA from New York, New York, and included 3 genome sequences from a previously published study (*14*) that reported identical or similar PFGE patterns and sequence types with those of the South Bronx hotel outbreak strain. These genomes differed by <5 SNPs from those of the South Bronx hotel isolates. The oldest *L. pneumophila* serogroup 1 isolate, dating back to 2007, had only 3 SNP differences, indicating that the isolate that caused the current outbreak had been present in the Bronx for >8 years. Two clinical isolates and 1 environmental isolate (NH1, NH2, and NH3) obtained during an outbreak in a Bronx nursing home in 2011–2012 were also found to be closely related to the South Bronx hotel isolate (<3 SNP differences), which indicated that this isolate caused >1 previous outbreaks of LD. WGS comparison of 4 other clinical isolates (09–214, 10–351, 10–423, and 10–458) from 2009 and 2010 showed an SNP profile that was identical to that of 3 of the clinical isolates from 2015 (15–157, 15–158, and 15–202) not epidemiologically linked to the South Bronx hotel–associated outbreak. These 2015 clinical isolates differed by 3 SNPs from the South Bronx hotel isolate, which suggested that patients might have been infected by an independent source that was not identified. In addition to SNPs, other genomic differences, such as the presence of plasmids or large indels, were also detected in some of the genomes analyzed.

WGS analysis of 6 *L. pneumophila* serogroup 1 isolates from a second, late summer outbreak in 2015 in the East Bronx neighborhood (15 cases, 4 clinical isolates, and 2 environmental isolate sequences) that was not suspected to be linked with the July outbreak, was confirmed to be unrelated (1,038 SNP differences) when compared with the South Bronx hotel outbreak strain. However, closer examination of locations of the SNPs showed that most differences were highly clustered in a few genomic locations, rather than being randomly dispersed throughout the genome, and might have been the result of recombination events. Only 8 SNP differences remained when these recombination locations were omitted. Clustering of SNPs in an otherwise isogenic background suggests that the East Bronx and South Bronx strains only recently diverged after horizontal gene transfer events. WGS was the only method powerful enough to discriminate between South Bronx hotel and all the other environmental isolates, including the homeless shelter, and confirmed the South Bronx hotel cooling tower as the source of this outbreak.

## Discussion

This outbreak investigation represents a large-scale testing effort by the NYC PHL, WC, and CDC public health laboratories. As reported by Weiss et al. (*9*), the environmental and epidemiologic investigation provided a comprehensive set of samples and specimens for laboratory testing.

Large outbreaks of LD can occur in areas of high population density that are near human-made reservoirs and mechanisms of aerosolization, such as cooling towers (*28*–*30*). Preventing or controlling such outbreaks in urban areas is further complicated by the presence of multiple potential reservoirs, which present substantial challenges when attempting to determine the exact point source. In a metagenomics survey of air samples obtained in New York, New York, *Legionella* was the predominant genus identified in samples collected from the rooftop of an office building overlooking midtown Manhattan (*31*). Further complicating epidemiology studies, it has been shown that aerosols containing *L. pneumophila* are capable of infecting persons residing at a distance of >6 km from the contaminated source (*32*).

Our findings of similar *L. pneumophila* strains at multiple locations and over extended periods is consistent with results of these studies and further suggest that *L. pneumophila* is capable of long-term survival in multiple reservoirs over large areas in an urban environment. Our findings also suggest that cooling towers colonized with *L. pneumophila* might contaminate other sites located nearby, leading to the possibility for an endemic strain to reestablish colonization after elimination of the organism at any single presumed source. This analysis warns us that because of the particular biologic and ecologic nature of *L. pneumophila*, reliance solely on 1 source of evidence (epidemiologic approaches or molecular data) might be insufficient to identify exact sources of legionellosis outbreaks.

Our extensive sampling and WGS of cooling tower isolates has shown that many cooling towers were colonized with a diverse and heterogeneous *Legionella* population, most of which have not caused detectable human disease. In the specific case of the South Bronx hotel cooling tower, 2 different *L. pneumophila* serogroup 1 strains were obtained (among 10 isolates recovered), including the strain responsible for the 2015 outbreak. This finding showed that populations of virulent clones can coexist among a wide variety of nonoutbreak strains not associated with known disease, and for which virulence has not been assessed.

It is still uncertain what triggered the LD outbreak in New York, New York, in 2015, but several factors might have contributed. Improper maintenance of cooling towers or excessive mist generated during operation could have created ideal conditions for *Legionella* spp. to multiply and aerosolize (*33*,*34*). In addition, a new *Legionella* subpopulation could have acquired, through mutation or recombination, new beneficial phenotypic capabilities (such as increased resistance to cleaning agents), better survival to desiccation, or enhanced aerosolization capability (*35*–*37*). The low level of heterogeneity seen between the historical and 2015 isolates is consistent with results from a similar study of a persistent *L. pneumophila* serogroup 1 outbreak–associated strain in Alcoy, Spain, where it was estimated that mutation rates for *L. pneumophila* in cooling towers can be as low as ≈0.15 SNPs/genome/year (or 1 mutation across the entire genome every 6.7 years) (*38*). This estimation raises the possibility that *L. pneumophila* can persist unchanged for extended periods in a dormant state until it is reactivated by favorable environmental conditions.

**Figure 4:**
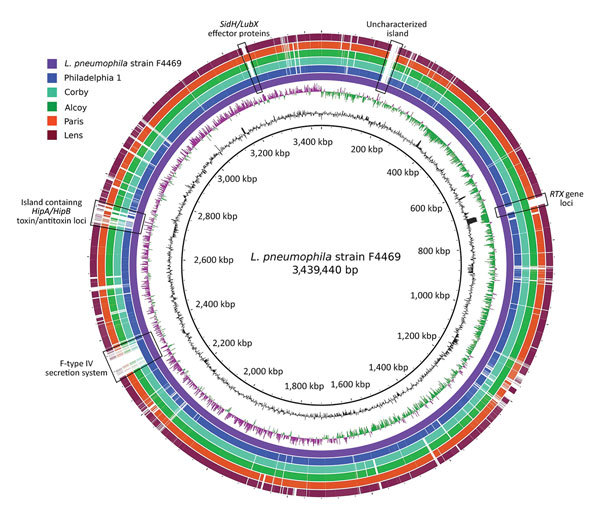
Blast ring genome comparison of *Legionella* *pneumophila* strains from investigation of Legionnaires’ disease outbreak caused by an endemic strain of *L. pneumophila*, New York City, New York, USA, 2015. Comparison is shown between South Bronx outbreak strain (F4469) and other sequenced strains (Philadelphia 1, Corby, Alcoy, Paris, and Lens). The 2 innermost circles indicate G + C content and G + C skew, respectively, of the outbreak strain genome. Gaps in outer circles indicate genome areas in strain F4469 that are either absent or of low identity in compared genomes. Most of these regions are composed of virulence factor–associated genes, such as an F-type IVA secretion system, effector protein genes, toxin/antitoxin loci, and genes with unknown functions. *Hip*, hippurate hydrolysis gene; *Lub*, *Legionella* U-box gene; *RTX*, repeats in structural toxin gene; *Sid*; substrate of macrophage killing/defective organelle trafficking transporter gene.

Genome analysis of the South Bronx outbreak strain identified several variable regions, many of which are associated with virulence factors, when compared with 5 previous outbreak-associated *L. pneumophila* strains (Figure 4). Two regions, 1 containing genes encoding an F-type IVA secretion system and 1 encoding *Legionella* U-box type E3 ligase/effector proteins, are also present in the 1976 Philadelphia 1 and Paris strains but absent from the other strains analyzed. The South Bronx outbreak strain F4469 also harbors an expanded isoform of the repeats in structural toxin gene (*rtxA*), similar to that found in the Corby and Alcoy strains. Finally, 2 genomic islands, 1 containing the hippurate hydrolysis A and B gene toxin–antitoxin system, as well as other hypothetical genes, and 1 containing mostly uncharacterized genes, were found to be unique to the South Bronx strain. BLAST (https://blast.ncbi.nlm.nih.gov/Blast.cgi) searches on these 2 regions found matches with partial homology with other *L. pneumophila* strains in the National Center for Biotechnology (Bethesda, MD, USA) nonredundant database. Further laboratory investigations will be required to determine the role of these islands, if any, to pathogenicity of this strain.

Our analysis showed the presence of *L. pneumophila* strain F4469 in the Bronx since 2007 at multiple locations associated with different outbreaks and sporadic LD. Although it is unclear what caused the identified cooling tower to contribute to so many cases, our findings suggest that a persistent and pathogenic endemic strain exists and might pose a risk for future outbreaks. Conventionally, cooling towers are believed to be seeded by municipal water distribution networks, and although this factor might be true, in a densely populated area such as New York, New York, cross-contamination between towers is a real possibility. This contamination can potentially lead to reestablishment of *L. pneumophila* in cooling towers after decontamination and cause long-term persistence of endemic strains in communities. Therefore, strict protocols regarding tower operation, maintenance, and cleanup, such as those mandated by recent New York State and New York City legislation, might help to minimize risks associated with locally circulating *L. pneumophila* strains (*39*,*40*).

## References

[1] Wallis L, Robinson P. Soil as a source of *Legionella pneumophila* serogroup 1 (Lp1). Aust N Z J Public Health. 2005;29:518–20. 10.1111/j.1467-842X.2005.tb00242.x 16366061

[2] Fields BS, Benson RF, Besser RE. *Legionella* and Legionnaires’ disease: 25 years of investigation. Clin Microbiol Rev. 2002;15:506–26. 10.1128/CMR.15.3.506-526.2002 12097254 PMC118082

[3] World Health Organization (WHO). Legionellosis, fact sheet no. Nov 2014 [cited 2017 Jul 20]. http://www.who.int/mediacentre/factsheets/fs285/en/

[4] Cunha BA, Burillo A, Bouza E. Legionnaires’ disease. Lancet. 2016;387:376–85. 10.1016/S0140-6736(15)60078-2 26231463

[5] Correia AM, Ferreira JS, Borges V, Nunes A, Gomes B, Capucho R, et al. Probable person-to-person transmission of Legionnaires’ disease. N Engl J Med. 2016;374:497–8. 10.1056/NEJMc1505356 26840151

[6] Fraser DW, Tsai TR, Orenstein W, Parkin WE, Beecham HJ, Sharrar RG, et al. Legionnaires’ disease: description of an epidemic of pneumonia. N Engl J Med. 1977;297:1189–97. 10.1056/NEJM197712012972201 335244

[7] McDade JE, Shepard CC, Fraser DW, Tsai TR, Redus MA, Dowdle WR. Legionnaires’ disease: isolation of a bacterium and demonstration of its role in other respiratory disease. N Engl J Med. 1977;297:1197–203. 10.1056/NEJM197712012972202 335245

[8] Mercante JW, Morrison SS, Desai HP, Raphael BH, Winchell JM. Genomic analysis reveals novel diversity among the 1976 Philadelphia Legionnaires’ disease outbreak isolates and additional ST36 strains. PLoS One. 2016;11:e0164074. 10.1371/journal.pone.0164074 27684472 PMC5042515

[9] Weiss D, Boyd C, Rakeman JL, Greene SK, Fitzhenry R, McProud T, et al.; South Bronx Legionnaires’ Disease Investigation Team. A large community outbreak of Legionnaires’ disease associated with a cooling tower in New York City, 2015. Public Health Rep. 2017;132:241–50. 10.1177/0033354916689620 28141970 PMC5349490

[10] Gaia V, Fry NK, Afshar B, Lück PC, Meugnier H, Etienne J, et al. Consensus sequence-based scheme for epidemiological typing of clinical and environmental isolates of *Legionella pneumophila.* J Clin Microbiol. 2005;43:2047–52. 10.1128/JCM.43.5.2047-2052.2005 15872220 PMC1153775

[11] Ratzow S, Gaia V, Helbig JH, Fry NK, Lück PC. Addition of *neuA*, the gene encoding N-acylneuraminate cytidylyl transferase, increases the discriminatory ability of the consensus sequence-based scheme for typing *Legionella pneumophila* serogroup 1 strains. J Clin Microbiol. 2007;45:1965–8. 10.1128/JCM.00261-07 17409215 PMC1933043

[12] Nazarian EJ, Bopp DJ, Saylors A, Limberger RJ, Musser KA. Design and implementation of a protocol for the detection of *Legionella* in clinical and environmental samples. Diagn Microbiol Infect Dis. 2008;62:125–32. 10.1016/j.diagmicrobio.2008.05.004 18621500

[13] Mérault N, Rusniok C, Jarraud S, Gomez-Valero L, Cazalet C, Marin M, et al.; DELPH-I Study Group. Specific real-time PCR for simultaneous detection and identification of *Legionella pneumophila* serogroup 1 in water and clinical samples. Appl Environ Microbiol. 2011;77:1708–17. 10.1128/AEM.02261-10 21193672 PMC3067292

[14] Raphael BH, Baker DJ, Nazarian E, Lapierre P, Bopp D, Kozak-Muiznieks NA, et al. Genomic resolution of outbreak-associated *Legionella pneumophila* serogroup 1 isolates from New York State. Appl Environ Microbiol. 2016;82:3582–90. 10.1128/AEM.00362-16 27060122 PMC4959152

[15] Chin C-S, Alexander DH, Marks P, Klammer AA, Drake J, Heiner C, et al. Nonhybrid, finished microbial genome assemblies from long-read SMRT sequencing data. Nat Methods. 2013;10:563–9. 10.1038/nmeth.2474 23644548

[16] Ribeiro FJ, Przybylski D, Yin S, Sharpe T, Gnerre S, Abouelleil A, et al. Finished bacterial genomes from shotgun sequence data. Genome Res. 2012;22:2270–7. 10.1101/gr.141515.112 22829535 PMC3483556

[17] Krumsiek J, Arnold R, Rattei T. Gepard: a rapid and sensitive tool for creating dotplots on genome scale. Bioinformatics. 2007;23:1026–8. 10.1093/bioinformatics/btm039 17309896

[18] Langmead B, Trapnell C, Pop M, Salzberg SL. Ultrafast and memory-efficient alignment of short DNA sequences to the human genome. Genome Biol. 2009;10:R25. 10.1186/gb-2009-10-3-r25 19261174 PMC2690996

[19] Li H, Handsaker B, Wysoker A, Fennell T, Ruan J, Homer N, et al.; 1000 Genome Project Data Processing Subgroup. The Sequence Alignment/Map format and SAMtools. Bioinformatics. 2009;25:2078–9. 10.1093/bioinformatics/btp352 19505943 PMC2723002

[20] GitHub. Bayesian haplotype-based genetic polymorphism discovery and genotyping [cited 2017 Aug 28]. https://github.com/ekg/freebayes

[21] Danecek P, Auton A, Abecasis G, Albers CA, Banks E, DePristo MA, et al.; 1000 Genomes Project Analysis Group. The variant call format and VCFtools. Bioinformatics. 2011;27:2156–8. 10.1093/bioinformatics/btr330 21653522 PMC3137218

[22] Li H, Durbin R. Fast and accurate long-read alignment with Burrows-Wheeler transform. Bioinformatics. 2010;26:589–95. 10.1093/bioinformatics/btp698 20080505 PMC2828108

[23] Francisco AP, Vaz C, Monteiro PT, Melo-Cristino J, Ramirez M, Carriço JA. PHYLOViZ: phylogenetic inference and data visualization for sequence based typing methods. BMC Bioinformatics. 2012;13:87. 10.1186/1471-2105-13-87 22568821 PMC3403920

[24] Bankevich A, Nurk S, Antipov D, Gurevich AA, Dvorkin M, Kulikov AS, et al. SPAdes: a new genome assembly algorithm and its applications to single-cell sequencing. J Comput Biol. 2012;19:455–77. 10.1089/cmb.2012.0021 22506599 PMC3342519

[25] Darling AC, Mau B, Blattner FR, Perna NT. Mauve: multiple alignment of conserved genomic sequence with rearrangements. Genome Res. 2004;14:1394–403. 10.1101/gr.2289704 15231754 PMC442156

[26] Mostowy R, Croucher NJ, Andam CP, Corander J, Hanage WP, Marttinen P. Efficient inference of recent and ancestral recombination within bacterial populations. Mol Biol Evol. 2017;34:1167–82. 10.1093/molbev/msx066 28199698 PMC5400400

[27] Alikhan N-F, Petty NK, Ben Zakour NL, Beatson SA. BLAST Ring Image Generator (BRIG): simple prokaryote genome comparisons. BMC Genomics. 2011;12:402. 10.1186/1471-2164-12-402 21824423 PMC3163573

[28] Jones KE, Patel NG, Levy MA, Storeygard A, Balk D, Gittleman JL, et al. Global trends in emerging infectious diseases. Nature. 2008;451:990–3. 10.1038/nature06536 18288193 PMC5960580

[29] Yang K, LeJeune J, Alsdorf D, Lu B, Shum CK, Liang S. Global distribution of outbreaks of water-associated infectious diseases. PLoS Negl Trop Dis. 2012;6:e1483. 10.1371/journal.pntd.0001483 22348158 PMC3279334

[30] Alirol E, Getaz L, Stoll B, Chappuis F, Loutan L. Urbanisation and infectious diseases in a globalised world. Lancet Infect Dis. 2011;11:131–41. 10.1016/S1473-3099(10)70223-1 21272793 PMC7106397

[31] Yooseph S, Andrews-Pfannkoch C, Tenney A, McQuaid J, Williamson S, Thiagarajan M, et al. A metagenomic framework for the study of airborne microbial communities. PLoS One. 2013;8:e81862. 10.1371/journal.pone.0081862 24349140 PMC3859506

[32] Nguyen TM, Ilef D, Jarraud S, Rouil L, Campese C, Che D, et al. A community-wide outbreak of legionnaires disease linked to industrial cooling towers—how far can contaminated aerosols spread? J Infect Dis. 2006;193:102–11. 10.1086/498575 16323138

[33] Mouchtouri VA, Goutziana G, Kremastinou J, Hadjichristodoulou C. *Legionella* species colonization in cooling towers: risk factors and assessment of control measures. Am J Infect Control. 2010;38:50–5. 10.1016/j.ajic.2009.04.285 19699013

[34] Garrison LE, Kunz JM, Cooley LA, Moore MR, Lucas C, Schrag S, et al. Vital signs: deficiencies in environmental control identified in outbreaks of Legionnaires’ disease—North America, 2000–2014. MMWR Morb Mortal Wkly Rep. 2016;65:576–84. 10.15585/mmwr.mm6522e1 27281485

[35] Dennis PJ, Lee JV. Differences in aerosol survival between pathogenic and non-pathogenic strains of *Legionella pneumophila* serogroup 1. J Appl Bacteriol. 1988;65:135–41. 10.1111/j.1365-2672.1988.tb01501.x 3204070

[36] D’Auria G, Jiménez-Hernández N, Peris-Bondia F, Moya A, Latorre A. *Legionella pneumophila* pangenome reveals strain-specific virulence factors. BMC Genomics. 2010;11:181. 10.1186/1471-2164-11-181 20236513 PMC2859405

[37] Cianciotto NP. Pathogenicity of *Legionella pneumophila.* Int J Med Microbiol. 2001;291:331–43. 10.1078/1438-4221-00139 11727817

[38] Sánchez-Busó L, Comas I, Jorques G, González-Candelas F. Recombination drives genome evolution in outbreak-related *Legionella pneumophila* isolates. Nat Genet. 2014;46:1205–11. 10.1038/ng.3114 25282102

[39] New York State Department of Health. Legionellosis (Legionnaires’ disease). May 11, 2016 [cited 2016 May 12]. https://www.health.ny.gov/diseases/communicable/legionellosis/

[40] New York City Department of Health and Mental Hygiene. Maintaining cooling towers. March 1, 2016 [cited 2016 May 12]. http://www1.nyc.gov/site/doh/business/permits-and-licenses/cooling-towers.page

